# Comprehensive insights into bone health, hormonal patterns, and fracture risks among men with drug resistant epilepsy: a crucial evaluation

**DOI:** 10.20945/2359-4292-2026-0082

**Published:** 2026-07-27

**Authors:** Cristian Pieper, Leonardo Vieira Neto, Eduardo Ferreira da Gama, Francisco P. Paranhos Neto, Miguel Madeira, Laura M. Carvalho Mendonça, Isabella D’Andrea Meira, Maria Lucia Fleiuss Farias

**Affiliations:** 1 Divisão de Endocrinologia, Universidade Federal do Rio de Janeiro, Rio de Janeiro, RJ, Brasil; 2 Centro de Epilepsia, Instituto Estadual do Cérebro Paulo Niemeyer, Rio de Janeiro, RJ, Brasil; 3 Divisão de Anestesiologia, Instituto Estadual do Cérebro Paulo Niemeyer, Rio de Janeiro, RJ, Brasil; 4 Divisão de Reumatologia, Universidade Federal do Rio de Janeiro, Rio de Janeiro, RJ, Brasil

**Keywords:** Epilepsy, antiseizure medications, bone mineral density, body composition

## Abstract

**Objective:**

To evaluate bone density and microstructure, fragility fractures, and hormones in men 50 years and older receiving antiseizure medications (ASMs).

**Subjects and methods:**

Study population included 39 men with difficult-to-control epilepsy and 42 healthy men as control group. Spine and hip areal bone mineral density (aBMD), body composition and vertebral fracture assessment (VFA) were evaluated using Dual-Energy X-Ray Absorptiometry (DXA). Volumetric bone mineral density (vBMD) and microarchitecture of trabecular and cortical compartments were evaluated using High-Resolution Peripheral Quantitative Computed Tomography (HR-pQCT). Physical activity was tested by the International Physical Activity Questionnaire (IPAQ). The fracture risk was estimated by fracture risk assessment tool (FRAX).

**Results:**

ASMs patients showed lower cortical bone thickness (Ct.Th) in distal tibia (1.196 ± 0.295 vs. 1.341 ± 0.241 mm, p = 0.03), more fractures (12 vs. 2, p = 0.003) mostly morphometric vertebral fractures, and a higher risk for major and hip fractures based on FRAX. Hormonal changes included higher median parathyroid hormone (PTH) (50.4 vs. 24 pg/mL, p < 0.001) possibly associated with vitamin D insufficiency, (25(OH)D levels below 30 ng/mL in 43.6% of ASMs patients), lower median Estradiol that was an independent factor for fractures (p = 0.04) and bioavailable Testosterone in the lowest quartile in 52 % of them.

**Conclusion:**

Men on ASMs exhibit lower Ct.Th and hormone changes that may contribute to bone fragility. While clinical fractures are infrequent, it is crucial to actively search for morphometric fractures, either during DXA or through the more available method of spine X-rays.

## INTRODUCTION

Pharmacoresistant epilepsy, which affects 30% of all individuals with epilepsy, presents a considerable challenge in epilepsy management. This condition is defined by the inability to secure sustained seizure control after adequate trials of two well-chosen, well-tolerated, and properly administered antiseizure medications (ASMs) schedules, whether as monotherapies or in combination (^1^). For such patients, the complexity of treatment increases, often necessitating the use of a combination of ASMs, aiming to maintain effectiveness while minimizing side effects. Studies have shown that ASMs that induce liver enzymes through cytochrome P450, such as carbamazepine, oxcarbazepine, phenobarbital, phenytoin, and topiramate, are more negatively associated with bone mineral density (BMD) than non-enzyme-inducing drugs like gabapentin, lacosamide, lamotrigine, levetiracetam, pregabalin, and valproate (^2^-^4^). Additionally, ASMs usage has been correlated with lowered levels of vitamin D and sex steroids, with restrictions on physical activity and the presence of sarcopenia potentially contributing to increased bone loss and fracture risks in this demographic (^5^,^6^).

The unpredictable nature of epileptic seizures elevates the risk of falls, significantly increasing the chance of fractures and adding to both the physical morbidity and the psychological and social impact on the patient (^5^,^7^). This underpins the importance of optimizing bone health within the epileptic community.

This study aimed to investigate bone density and microstructure, physical activity, the occurrence of fragility fractures, and the hormonal profiles in men aged 50 and above with epilepsy who are on ASMs therapy.

## SUBJECTS AND METHODS

### Study design and patient selection

This observational, cross-sectional study was performed in a single referral center for the treatment of epilepsy in southern Brazil, the Instituto Estadual do Cérebro Paulo Niemeyer (IECPN). The inclusion criteria were male adults 50 years and older with epilepsy who had been treated at IECPN outpatient clinic for at least two years. Women were excluded from this study because menopause would interfere with interpretation of bone events. The following risk factors for the development of osteoporosis were excluding criteria: vegetarian or vegan diet; regular use of medications that might interfere with bone homeostasis, such as glucocorticoids, sex hormones, antidepressants, proton pump inhibitors, antipsychotics, as well as antiosteoporosis drugs; malignant neoplasia in the previous five years; chronic diseases affecting the kidneys or the respiratory or cardiac systems; cerebral palsy; and any condition favoring immobility. Patients with a body mass index (BMI) below 18 kg/m^2^ or above 40.0 kg/m^2^ were also excluded. The control group was composed of healthy men 50 years and older, recruited from family members or friends of patients who participated in research studies including DXA and HRPQCT evaluation that were developed in the osteometabolism unit of the Endocrinology Division of HUCFF-UFRJ. The present study was approved by the local research ethics committee (CAAE: 39122720.1.0000.8110), and all participants signed an informed consent form before being included in the study. Laboratory and imaging tests were collected at a single time point.

### Data collection

All participants completed a questionnaire that addressed their medical history, information about previous fractures and also parental hip fractures, falls and injuries; smoking; alcoholism; data on physical activity considered in IPAQ; and intake of dairy products and vitamin D or calcium supplements. Demographic data on weight (W) and height (HT), which were used to calculate BMI (BMI=W/HT^2^), as well as age and other variables, were also collected.

### Laboratory

Blood samples were drawn between 8 AM and 10 AM after an overnight fast. Glucose (reference values: 60-99 mg/dL), calcium (8.5-10.1 mg/dL), phosphorus (3.0-4.5 mg/dL), albumin (3.5-5.5 g/dL), gamma glutamyl transferase (GGT; 12-45 U/L), and creatinine (0.8-1.3 mg/dL) were measured using colorimetric methods. The glomerular filtration rate (GFR) was estimated using the chronic kidney disease epidemiology collaboration equation (^8^). Chemiluminescent assays using commercial kits Ebran^®^ were used to measure total alkaline phosphatase (ALP; 27-100 U/L) and the following hormones: free thyroxine (FT4; 0.54-1.24 ng/dL), thyrotropin (TSH; 0.38-5.33 µUI/mL), 25-hydroxyvitamin D (25(OH)D; 20-60 ng/mL), intact parathyroid hormone (PTH; 12-88 pg/mL), estradiol (E2; < 33 pg/mL), sex hormone-binding globulin (SHBG; 13.2-89.5 nmol/L), and testosterone (T; 175-761 ng/dL). Bioavailable testosterone (Bio T; 50-59 years: 99.3-288.3 ng/dL, 60-69 years: 84.1-269.9 ng/dL, 70-79 years: 74.6-251.4 ng/dL) was calculated based on total T, SHBG, and albumin using a validated equation (^9^).

### Physical Activity Questionnaire (IPAQ)

Physical activity practice was estimated using the International Physical Activity Questionnaire (IPAQ), a method that has been validated for the Brazilian population, including elderly males (^10^). The participants were asked about the frequency and duration of the activities in which they engaged in a typical week. The responses were used to categorize the participants into the following three groups based on their level of physical activity: inactive, active, and very active.

**Dual-Energy X-Ray Absorptiometry (DXA**)

Areal bone mineral density (aBMD), body composition and vertebral fracture assessment (VFA) were carried out on all participants using the same equipment Prodigy Advanced Plus, GE-Lunar, EUA. BMD (g/cm^2^) was measured at lumbar spine (LS), femoral neck (FN), and total hip (TH). The T-score is defined as the number of standard deviations from the patient’s BMD and the mean reference value of the normal young population. Based on their lowest T-score, the participants were classified as having osteoporosis (T ≤ -2.5 SD), low BMD or osteopenia (T-score between < -1 and > -2.5 SD), or normal BMD (T ≥ -1 SD). The precision error (coefficient of variation, %CV) and the least significant change (LSC = 2.77 x precision error) of the DXA Prodigy Advance were calculated based on the ISCD (International Society of Clinical Densitometry) orientation. Thirty patients (age range 20-65 years) were scanned twice for spine, hip and total body. After the first scan, each patient was removed from the table and repositioned for the second scan. All measurements were entered in the equation available on the ISCD site https://iscd.org/resources/calculators/. Results obtained for the Root Mean Square Standard Deviation (RMS-SD) and the LSC CI 95% were 0.010 g/cm^2^ and 0.028g/cm^2^ for lumbar spine, and 0.012 g/cm^2^ and 0.034 g/cm^2^ for total hip. For longitudinal studies, we assume changes at or higher than 1.5% for the lumbar spine and 2.3% for the total hip as significant.

Body composition provided data on fat and lean mass. To estimate muscle mass, appendicular lean mass (ALM; lean mass in the arms and legs) was calculated and adjusted for BMI and height (ALM/BMI and ALM/HT^2^, respectively).

Non-clinical vertebral fractures were actively sought via VFA using a visual scale to detect deformities compatible with morphometric vertebral fractures. All exams were conducted and evaluated by a professional with extensive experience in densitometry, according to the International Society for Clinical Densitometry guidelines (^11^).

### FRAX

The fracture risk assessment tool (FRAX), which has been validated for the Brazilian population (^12^), was employed to estimate the probability of hip and/or major osteoporotic fractures (which include hip, vertebrae, distal forearm, and proximal humerus fractures) in the next 10 years of life.

### High-Resolution Peripheral Quantitative Computed Tomography (HR-pQCT)

Volumetric bone density and microarchitecture of the trabecular and cortical compartments were evaluated at the distal radius (non-dominant arm) and distal tibia using a first-generation high-resolution peripheral quantitative computed tomography (HR-pQCT) system called Xtreme CT I (Scanco Medical AG, Brüttisellen, Switzerland). From both sites, 110 slices with a nominal resolution (voxel size) of 82 µm were obtained. The first slice was 9.5 mm and 22.5 mm proximal to the reference line for the distal radius and distal tibia, respectively. The parameters obtained were nominated following the recommendations of a recent consensus (^13^). Volumetric bone density was measured at the entire bone [Tt.BMD], cortical bone [Ct.BMD] and trabecular bone [Tb.BMD]. Microstructural parameters included: cortical thickness [Ct.Th: ratio of cortical bone volume to the outer bone surface], trabecular number [Tb.N], trabecular bone volume fraction [BV/TV, derived from trabecular density/1200 mg hydroxyapatite], trabecular thickness [Tb.Th:(BV/TV)/Tb.N], trabecular separation [Tb.Sp: (1-BV/TV)/Tb.N], and trabecular inhomogeneity [Tb.1/N.SD, the standard deviation of 1/Tb.N, which reflects the inhomogeneity of the network]. Unlike DXA, there are no standardized values for healthy young people regarding volumetric density or microstructure parameters. Therefore, the exam does not define T-scores or Z-scores.

Quality control included daily phantom measurements to ensure the stability of volumetric density measurements (QC1), and weekly phantom assessments were conducted to verify long-term scanner stability and consistency of structural measurements (QC2). Patients positioning, movement artifacts and image examination were performed and analyzed by the same experienced professional according to the manufacturer’s recommendations and described in detail by Whittier and cols. (^13^).

There are few reports on CV% RMS and LSC values, but acceptable values for CV% widely cited for the X-treme CT1 reproducibility were described by Mikolajewicz and cols. (^14^), and vary from 0.8-2.0% for vBMD, 2.3-4.0% for bone area and 1.6-4.9% for trabecular and cortical microstructure. The HR-pQCT evaluation allowed to differentiate patients with and without fractures, despite only some of the measures were significantly larger than the LSC (Tt.vBMD; Tb.vBMD; Ct.Th and Ct.vBMD at the tibia only.

Considering the difficulty to estimate CVs for every HR-pQCT parameter, we assume, for longitudinal studies, CV values from 1-2% for density measurements and 3-5% for microarchitectural parameters.

### Statistics

The statistical analyses were performed using SPSS version 23.0 for MacOS (SPSS Inc., Chicago, IL, USA). In the descriptive analysis, the categorical variables were expressed as frequency and percentage, and the numerical variables were expressed as mean ± SD. The Kolmogorov-Smirnov test defined the variables with a normal distribution. The student’s t-test or the Mann-Whitney U test was performed to compare the numerical variables between the groups. The chi-square test or the Fisher’s exact test was used to compare the categorical variables. Multivariate analysis was performed to test the contribution of PTH, Estradiol, Cortical bone thickness and IPAQ in the occurrence of fragility fractures. A *p*-value < 0.05 was considered significant.

## RESULTS

**Figure 1:**
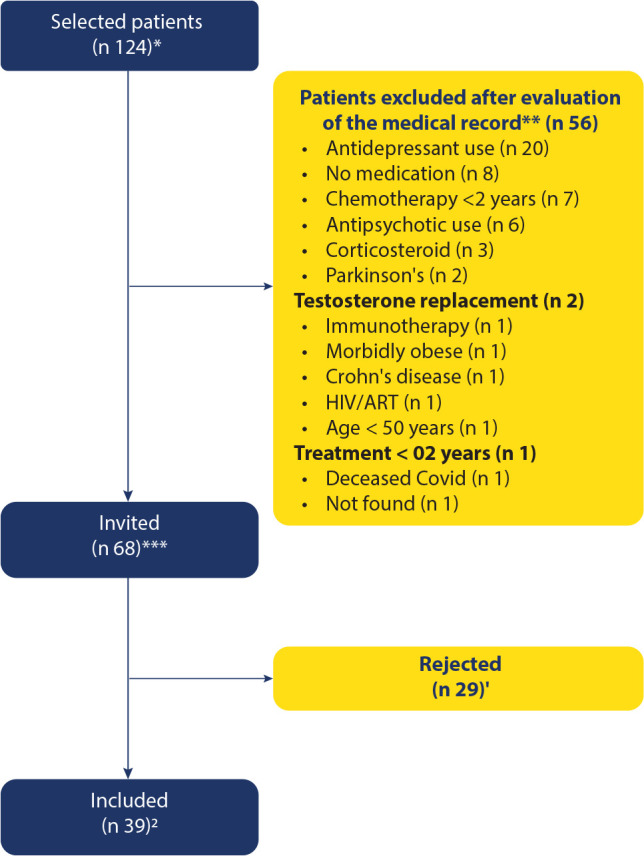
Flow chart of patient’s selection

The medical records of 124 male epileptic patients treated at the IECPN outpatient clinic between January and December 2021 were screened for inclusion in this study. After initial analysis, 56 patients were excluded based on predefined exclusion criteria. Subsequently, the remaining 68 patients were contacted by phone and invited to participate in the study. Of these, 39 completed all assessments as outlined in the study protocol (see **Figure 1**). The control group consisted of 45 healthy men. No significant differences were observed between the ASMs and control groups in terms of age, BMI, or the prevalence of type 2 diabetes mellitus (12.7% of the ASMs group and 16.3% of the control group) treated only with metformin.

*Selected medical records of male patients >50 years of age treated at the IECPN epilepsy outpatient clinic from January to December 2021. **Exclusion criteria found, ***Number of individuals invited to participate via telephone. ^1^ Individuals who refused to participate. ^2^ Individuals included in the survey after signing the TCLE.

**Figure 2:**
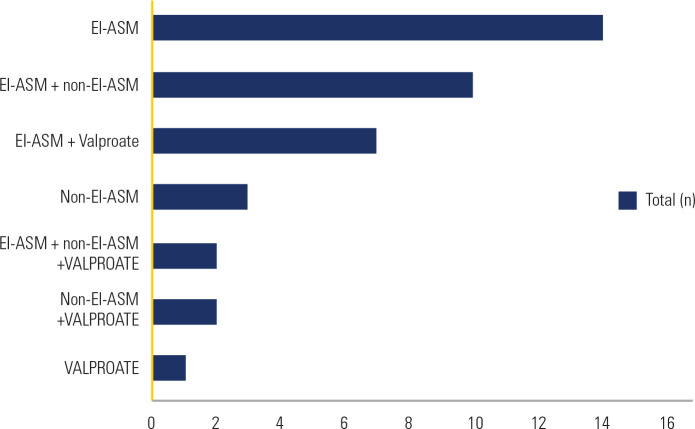
Combination of antiseizure medications

All the Study patients included had drug-resistant epilepsy and commonly required a combination of both enzyme-inducing antiseizure medications (EI-ASMs) (e.g., carbamazepine, oxcarbazepine, phenobarbital, phenytoin, topiramate) and non-enzyme-inducing antiseizure medications (non-EI-NASMs) (e.g., lamotrigine, levetiracetam, valproate). Specifically, 84.6% of patients were treated with EI-ASMs, 30.7% received valproate, and 43.5% were prescribed non-EI-ASMs. Additionally, 64% of patients used multiple drugs to manage their seizures (**Figure 2**). The ASMs treatment varied from 2 to 60 years, median duration 9 years. The etiology of epilepsy was most commonly observed were benign CNS tumors, followed by vascular causes and mesial temporal sclerosis (MTS). Imaging exams revealed nonspecific changes unrelated to epilepsy in six patients and no changes in three patients.

n: number of participants in each drug association; El-ASMs: enzyme-inducing antiseizure medications (Carbamazepine, Oxcarbazepine, Phenobarbital, Phenytoin and Topiramate); non-El-ASMs: non-enzyme-inducing antiseizure medications.

Regarding metabolic parameters all participants had serum calcium, phosphorus, creatinine, TSH, and FT4 levels within normal limits, with GFR at or above 60mL/min/1.73 m^2^. In comparison with controls, ASMs patients had higher GFR and liver enzymes (total alkaline phosphatase and GGT).

The 25(OH)D levels were below 20 ng/mL in 18% of the ASMs group and 9% of the control group. No participant had 25(OH)D values below 12 ng/mL, consistent with severe vitamin D deficiency. Serum PTH levels were higher in the ASMs patients.

Total testosterone levels were within normal limits for all participants, and the median values were even higher in the ASMs group. SHBG levels, only measured in ASMs patients, was in the highest quartile (above 70 nmol/L) in 34.3% of them. As a consequence, bioavailable testosterone was in the lowest quartile in 86% and below the age-adjusted reference values in 22% of them. Estradiol was significantly lower in the ASMs group than in the control group. These results are presented in **Table 1**.

**Table 1:** Comparison of biochemical data between epileptic patients and controls

	ASM (N = 39)	Control (N = 42)	P value
Age (years)	56 (54; 67)	58 (55.3; 64)	0.98
Height (cm)	170 (159; 183)	170 (150; 183)	0.99
Weight (kg)	78.3 (62.2; 118.7)	77.98 (56; 118)	0.91
BMI (kg/m^2^)	27.1 ± 3.4	27.0 ± 4	0. 98
GFR (mL/min/1.73 m^2^)	95.9 ± 16.4	86.1 ± 12.1	0.004
Calcium (mg/dL)	9.5 (9.1; 10)	9.2 (8.8; 9.5)	0.01
Phosphorus (mg/dL)	3.5 ± 0.8	3.4 ± 0.5	0.50
Albumin (g/dL)	4.2 ± 0.4	4.1 ± 0.3	0.10
GGT (U/L)	58 (32; 87)	33 (25; 56)	0.026
ALP (U/L)	175.5 ± 86.2	94.6 ± 44.6	0.026
25(OH)D (ng/dL)	30.4 (23.6; 42)	26 (22.7; 29.2)	0.009
25(OH)D below 20 ng/mL (%)	18	9.3	0.44
PTH (pg/mL)	50.4 (37.2; 68.6)	24 (17.9; 28.8)	<0.001
Total Testosterone (ng/dL)	402.5 ± 147.2	323.1 ± 108.6	0.026
Estradiol (pg/mL)	25 (18; 31)	49.7 (37; 63)	<0.001
FT4 (ng/dL)	0.89 ± 0.2	1.08 ± 0.15	0.02
TSH (µUI/mL)	2.0 (1.4; 2.7)	1.8 (1; 2.1)	0.28

Values are expressed as mean ± SD or median (1^st^-3^rd^ quartiles). BMI (body mass index), GFR (glomerular filtration estimated by CKD-EPI equation), GGT (gamma glutamyl transferase), 25(OH) (25-hydroxyvitamin D), PTH (parathyroid hormone), ALP (alkaline phosphatase FT4 (free thyroxine), TSH (thyroid stimulating hormone).

According to the IPAQ results, the ASMs group was less active than the control group. A comparison of the body composition of the groups revealed no significant difference in lean mass, which mostly reflected the muscle mass of the total body, arms, and legs. These results are shown in **Table 2**.

**Table 2:** Comparison of body composition and physical activity between epileptic patients and controls

	ASM (N = 39)	Control (N = 42)	P value
Total body fat mass (%)	32.47 ± 7.39	31.29 ± 4.43	0.42
Total body lean mass (kg)	50.64 ± 5.43	51.26 ± 7.67	0.71
Arms lean mass (kg)	6.26 ± 0.94	6.64 ± 1.16	0.16
Legs lean mass (kg)	17.06 ± 2.47	17.03 ± 3.61	0.96
Appendicular lean mass^*^	23.31 ± 3.23	23.67 ± 4.61	0.72
ALM/H^2^	8.08 ± 0.87	8.28 ± 1.37	0.49
ALM/BMI	0.03 ± 0.01	0.03 ± 0.01	0.28
IPAQ (%)^**^			0.003
Group 1 (high activity)	14.3	46.2	
Group 2 (moderate activity)	37.1	42.3	
Group 3 (low activity)	48.6	11.5	

Values expressed as mean ± SD or number of subjects - n (%),

* Lean mass in the arms and legs (ALM), ALM/BMI (adjusted for BMI), ALM/H^2^ (adjusted for height)

** IPAQ (International Physical Activity Questionnaire).

There was no difference in the frequence of normal BMD, low BMD (osteopenia), and osteoporosis between ASMs patients (58%, 39%, and 3%) and control (52%, 48%, and 0%), respectively. No statistically significant differences were found in areal BMD at the lumbar spine or hip between these groups (**Table 3**).

**Table 3:** Comparison of bone densitometry and fractures between epileptic patients and controls

	ASM (N = 39)	Control (N = 42)	P value
Lumbar spine BMD (g/cm^2^)	1.183 (1.060; 1.243)	1.172 (1.100; 1.301)	0.59
Femoral neck BMD (g/cm^2^)	0.953 (0.924; 1.135)	0.975 (0.916; 1.050)	0.21
Total hip BMD (g/cm2)	1.047 (0.766; 1.252)	1.060 (0.996; 1.110)	0.45
Patients with fractures: N (%)	12 (30.8)	2 (4.7)	0.003
FRAX major fractures (absolute risk)^1^	2.00 (1.60; 3.10)	1.85 (1.60; 2.13)	0.019
FRAX hip fractures (absolute risk)^2^	0.30 (0.10; 1.00)	0.20 (0.10; 0.50)	0.023

Values are expressed as Median (1^st^-3^rd^ quartiles) or number of subjects - n (%); BMD (areal bone mineral density);

1 FRAX assessment thresholds for ten-year probability of a major osteoporotic fracture,

2 FRAX assessment thresholds for ten-year probability of hip fracture.

Fractures were more frequent in the ASMs group; five referred prevalent fractures (three at the radius and two at the clavicle), and seven were diagnosed with morphometric vertebral fractures using VFA. Meanwhile, only two participants in the control group had morphometric vertebral fractures. These findings were consistent with the FRAX results, which showed that the ASM group had a higher risk of major osteoporotic and hip fractures than the control group (**Table 3**).

The HRpQCT results showed that the ASM group had lower Ct.Th at the distal tibia than the control group. No significant difference was detected in the trabecular compartment (**Table 4**).

**Table 4:** Comparison of bone density and microstructure evaluated by HR-pQCT between epileptic patients and controls

	ASM group (N = 39)	Control group (N = 42)	P value
Distal radius			
Tt.BMD (mg HA/cm^3^)	339.07 ± 78.20	328.98 ± 47.52	0.51
Ct.BMD (mg HA/cm^3^)	895.87 ± 75.15	869.14 ± 44.49	0.07
Ct.Th (mm)	0.860 ± 0.243	0.834 ± 0.183	0.61
Tb.BMD (mg HA/cm^3^)	176.63 ± 37.27	180.00 ± 26.29	0.66
BV/TV (^1^)	0.147 ± 0.031	0.150 ± 0.022	0.66
Tb.N (mm^-1^)	1.961 ± 0.282	2.055 ± 0.246	0.14
Tb.Th (mm)	0.075 ± 0.016	0.074 ± 0.012	0.92
Tb.Sp (mm)	0.444 ± 0.075	0.418 ±0.055	0.09
Tb.1/N.SD (mm)	0.200 ± 0.065	0.188 ± 0.062	0.43
Distal tíbia			
Tt.BMD (mg HA/cm^3^)	294.71 ± 59.01	302.20 ± 46.01	0.55
Ct.BMD (mg HA/cm^3^)	897.07 ± 57.17	895.38 ± 40.87	0.88
Ct.Th (mm)	1.196 ± 0.295	1.341 ± 0.241	0.03
Tb.BMD (mg HA/cm^3^)	165.74 ± 31.11	161.70 ± 33.75	0.61
BV/TV (^1^)	0.138 ± 0.026	0.135 ± 0.028	0.62
Tb.N (mm^-1^)	1.909 ± 0.306	1.920 ± 0.327	0.88
Tb.Th (mm)	0.073 ± 0.014	0.070 ± 0.009	0.27
Tb.Sp (mm)	0.464 ± 0.082	0.466 ± 0.093	0.94
Tb.1/N.SD (mm)	0.214 ± 0.049	0.214 ± 0.055	0.97

Values expressed as mean ± SD, BMD: bone mineral density; Tt.BMD (vBMD of entire bone), Ct.BMD (cortical vBMD), Ct.Th (cortical thickness), Tb.BMD (trabecular vBMD), BV/TV (bone volume-to-total volume ratio), Tb.N (trabecular number), Tb.Th (trabecular thickness), Tb.Sp (trabecular separation), Tb.1/N.SD (inhomogeneity of trabecular network).

Multivariate analysis identified Estradiol as an independent and significant factor for fractures (p=0.04), and PTH influence tended towards significance (p = 0.07), while Ct.Th and IPAQ were not associated with fractures.

## DISCUSSION

This study identified a higher prevalence of clinical fractures and morphometric vertebral fractures in men with difficult-to-control epilepsy as compared to controls, unrelated to decreased bone mineral density. Lower cortical bone thickness detected by HR-pQCT and hormonal changes may contribute to the bone fragility.

The predominant use of EI-ASMs arises from the fact that this class of medication is available free of charge in our public health system, enabling access to treatment for patients with low socioeconomic status. Chronic ASMs use frequently causes asymptomatic or transient elevations of liver enzymes, which have been described in 75-95% of patients (^15^,^16^). According to the authors, patients chronically treated with EI-ASMs showed high ALP activity, which could correspond to increase in bone isoenzyme and reflect clinical or subclinical osteomalacia, or it might have hepatic origin and reflect hepatic induction, not necessarily hepatocyte injury (^17^). The ASM group we studied had higher levels of liver enzymes, although the bone ALP isoenzyme was not measured. ASM group also showed lower FT4 despite normal TSH, which is in accordance with literature (^18^) and do not indicate functional hypothyroidism or have a negative influence on bone metabolism.

Bone health is often compromised (^19^), although mechanism is not fully understood. However, an established hypothesis is that ASMs such as carbamazepine, phenytoin, and oxcarbazepine cause hepatic enzyme induction via cytochrome P450, resulting in vitamin D catabolism and disturbances in sex steroids associated with increased SHBG concentrations (^20^).

Regarding bone health, ideal levels of vitamin D should guarantee maximum intestinal calcium absorption and adequate bone mineralization and prevent secondary hyperparathyroidism. An Institute of Medicine report (^21^) concluded that 25(OH)D levels ≥ 20 ng/mL are adequate for most people. In contrast, the Endocrine Society (^22^) asserted that the ideal level (especially for people at risk of fractures) is ≥ 30 ng/mL (vitamin D sufficiency) and that a level between 20 and 30 ng/mL corresponds to vitamin D insufficiency and a level <20 ng/mL represents vitamin D deficiency.

In the present study, none of the participants had severe vitamin D deficiency, as defined by 25(OH)D levels below 10-12 ng/mL (^23^), or clinical manifestations of osteomalacia (e.g., diffuse bone pain, skeletal deformities, pseudofractures) (^24^). However, considering that ASM patients are at risk of osteoporosis, the ideal vitamin D level should not be lower than 30 ng/mL, which occurred with 17 (43.6%) of them. This probably contributed to decreased intestinal calcium absorption and higher PTH levels in ASM patients. We cannot exclude they might have lower calcium intake, but it is worth note that all these disturbances favor secondary hyperparathyroidism (^25^,^26^). The negative impact of chronic hyperparathyroidism on bone density, quality, and fractures is well recognized. Bone resorption exceeds bone formation, leading to a negative imbalance in bone turnover and bone loss, mostly in the cortical compartment (^27^). A study of 287 elderly Brazilian women identified a significant association between vitamin D deficiency, cortical bone loss, and fragility fractures (^28^). It is worth note that alterations in cortical bone have been associated with the severity of vertebral fractures (^29^) and that ASM group had decreased cortical thickness at the distal tibia and vertebral fractures.

Regarding sex steroids, it is worth mentioning that estradiol is the most important regulator of bone remodeling, including in men (^30^). The conversion of androgens to estrogens is the major source of circulating estrogens in men and depends on cytochrome P450 aromatase. This enzymatic reaction occurs in adipose tissue and other tissues including brain and bone cells, and the local concentration of estradiol seems to contribute significantly to skeletal homeostasis (^31^). We found decreased estradiol in ASM patients, probably related to lower aromatase activity. On the other hand, the decreased conversion might explain higher total testosterone levels in ASM patients. Meanwhile, the modulation of androgen action depends on SHBG, as an increase in sex steroids binding decreases the free and bioavailable fractions of testosterone. Age-related increase in SHBG and decrease in bioavailable testosterone and estradiol were associated to bone loss in Brazilian men (^32^) and also with fragility fractures in elderly men (^33^). Furthermore, only higher SHBG (but not decreased sex steroids) was associated with fractures in a large cohort described by Cawthon and cols. (^34^). We found bioavailable testosterone below the normal reference values in 22% of the ASM patients and in the lower 1st-2nd quartiles in 86%. The discrepancy between total and bioavailable testosterone in epileptic patients using ASMs has been attributed to an increase in SHBG (^35^,^36^). SHBG was not evaluated in the controls, but SHBG levels were in the highest quartile in about one third of ASM patients. Thus, these hormonal changes probably contributed to fractures in ASM patients.

Drug-resistant epilepsy significantly compromises the quality of life and restricts usual activities. Regarding the assessment of body composition, previous studies have used different methods and obtained varied results. Szałwińska and cols. (^37^) used bioelectrical impedance to study 60 epileptic patients (18-73 year old) who had at least one year of treatment but did not find significant differences in body mass, lean body mass, free fat mass, total fat, or visceral fat when compared to a control group. Sarangi and cols. (^38^) also evaluated epileptic patients using bioelectrical impedance and found no significant differences between any of the study groups in terms of body composition parameters, even when the parameters were compared based on new and old ASMs. In the present study, all participants were evaluated for body composition using DXA, the most recommended method for this purpose. Despite being less active according to IPAQ evaluation, ASM patients did not show alteration in body composition. Although they were not evaluated with test for muscle function, the measurement of total and appendicular lean mass did not suggest sarcopenia in ASM patients (^39^). Furthermore, there were no differences in body fat mass which could influence the conversion of testosterone into estradiol.

People with epilepsy are at risk of developing osteoporosis (^4^), and prolonged ASM use is a risk factor that contributes to this condition (^40^). Shiek Ahmad and cols. (^41^) described changes in BMD in pairs of 48 same-sex twin/age-matched sibling that were discordant regarding the use of ASM. Compared with non-IE-ASM users, ASM users had lower FN, TH, and trochanter BMD. In addition, ASM users had higher rates of TH and whole-body bone loss per year than non-users. In the present study, about 50% of the ASM and control groups had normal BMD, and only 3% of the ASM group was classified as having osteoporosis based on DXA. Thus, bone density added little information on bone fragility associated with fractures

Compared to the general population, the risk of fractures was described as 2 to 6 times higher according to Petty and cols. (^42^). Falls during seizures and imbalances caused by ASM side effects result in increases in forearm and hip fractures in patients with epilepsy. The hip may be at greater risk due to a fall pattern in which patients may not be able to use their arms protect themselves. Crisis-related fractures represent only 25% to 43% of all fractures, indicating that other factors also contribute to increased risk (^43^-^45^). In a meta-analysis, Vestergaard (^46^) evaluated the effects of epilepsy on changes in BMD and the risk of fractures, concluding that low BMD in patients with epilepsy did not sufficiently explain the increased risk of fractures and suggesting that the involvement of microarchitectural changes could lead to a reduction in bone strength. Rolvien and cols. (^47^) used DXA and HR-pQCT to perform a skeletal assessment of a six-year-old girl with epileptic encephalopathy accompanied by severe juvenile osteoporosis and multiple skeletal fractures and found combined trabecular and cortical bone loss. We did not find studies of HR-pQCT in epileptic males. Thus, our study is the first to demonstrate cortical bone changes in a group of ASM men, who also presented increased serum PTH and decreased bioavailable testosterone and estradiol, and more fractures than control group, even considering only non-clinical vertebral fractures, that were unrelated to falls (18% in ASM and 4.7% in controls).

We recognize as the main limitation the small number of ASM patients included, which limits the generalizability of the results. Meanwhile, the strengths of the study included the careful selection of the sample, which enabled the creation of a homogeneous group of patients; stability in terms of the use of medications for at least two years; and the exclusion of important confounding factors, such as menopause. Although it was not possible to test associations between bone, ASMs and hormones due to the small sample size, we considered the contribution of calciotropic hormones and cortical bone changes to the higher prevalence of fractures, especially morphometric vertebral fractures, which were not associated with falls.

In conclusion, people with epilepsy should be examined for fractures and hormonal changes. In most cases, there is no clinical fracture, and a morphometric fracture is present. Although studies can be conducted using VFA, spine X-rays may be more adequate due to cost and accessibility, since DXA contributed little to the assessment of bone fragility. Overall, the active investigation of morphometric fractures is recommended for ASM males 50 years and older to help identify patients with bone fragility and start anti-osteoporosis medications.

## Data Availability

datasets related to this article will be avail-able upon request to the corresponding author.
